# Tumor vascular endothelial cells promote immune escape by upregulating PD-L1 expression via crosstalk between NF-κB and STAT3 signaling pathways in nasopharyngeal carcinoma

**DOI:** 10.1038/s41419-025-07444-z

**Published:** 2025-02-25

**Authors:** Yan Wang, Yuanyuan Chen, Yuanyuan Liu, Jingjing Zhao, Gongming Wang, Hao Chen, Yan Tang, Dijun Ouyang, Songzuo Xie, Jinqi You, Xinyi Yang, Minxing Li, Jianchuan Xia, Tong Xiang, Desheng Weng

**Affiliations:** 1 Department of Biotherapy, State Key Laboratory of Oncology in South China, Collaborative Innovation Center for Cancer Medicine, Guangdong Key Laboratory of Nasopharyngeal Carcinoma Diagnosis and Therapy, Guangdong Provincial Clinical Research Center for Cancer, Sun Yat-sen University Cancer Center, 510060 Guangzhou, China; 2 Department of Medical Oncology, The Third Affiliated Hospital of Sun Yat-sen University, 510630 Guangzhou, China; 3 Department of Thoracic Surgery, State Key Laboratory of Oncology in South China, Collaborative Innovation Center for Cancer Medicine, Guangdong Key Laboratory of Nasopharyngeal Carcinoma Diagnosis and Therapy, Guangdong Provincial Clinical Research Center for Cancer, Sun Yat-sen University Cancer Center, 510060 Guangzhou, China; 4 Department of Experimental Research, State Key Laboratory of Oncology in South China, Collaborative Innovation Center for Cancer Medicine, Guangdong Key Laboratory of Nasopharyngeal Carcinoma Diagnosis and Therapy, Guangdong Provincial Clinical Research Center for Cancer, Sun Yat-sen University Cancer Center, 510060 Guangzhou, China

**Keywords:** Prognostic markers, Cancer microenvironment

## Abstract

Aberrant vascular systems are significant indicators of cancer and play pivotal roles in tumor immunomodulation. However, the role of PD-L1 expressed on vascular endothelial cells (VECs) in the tumor immune microenvironment of nasopharyngeal carcinoma (NPC), as well as its correlation with patient prognosis, remains unclear. According to in vitro experiments conducted in our research, NPC tumor supernatants could upregulate PD-L1 expression on HUVECs, and the upregulated PD-L1 could bind to PD-1 on T cells leading to diminished T cell killing. The results of animal experiments similarly showed that elevated levels of PD-L1 on tumor VECs hindered the anti-tumor effectiveness of T cells, resulting in immune evasion and tumor progression. Furthermore, PD-L1 expression on tumor VECs served as a valuable prognostic marker, with heightened expression linked to poorer prognosis in NPC patients. Mechanistically, we discovered that the interaction between NF-κB and STAT3 signaling pathways may contribute significantly to the up-regulation of PD-L1 on VECs in NPC. Together, our work provides novel insights into identifying prognostic markers and strategies for reversing immune evasion mechanisms in NPC.

## Introduction

Nasopharyngeal carcinoma (NPC) is a unique type of head and neck cancer with a highly uneven geographical distribution, particularly prevalent in East and Southeast Asia, especially in Southern China [1, 2]. Etiologically, NPC is deeply associated with Epstein-Barr virus (EBV) infection, and its pathology is characterized by poorly differentiated cancer cells and a high degree of immune-cell infiltration within the tumor lesion [3, 4]. Typically, a high degree of immune-cell infiltration is associated with a better prognosis for patients. However, despite aggressive treatment, disease recurrence occurs in 5%–15% of patients, and distant metastases occur in 15%–30% of NPC patients [1, 5, 6]. In addition, approximately 6%–15% of NPC patients present with metastases at initial diagnosis [7]. The median progression-free survival (PFS) for these patients is approximately 7 months, with a median overall survival (OS) of less than 2 years [8]. Despite the heavy infiltration of immune cells, they may not effectively perform their normal functions.

The tumor microenvironment is an intricate milieu, an “ecological” system conducive to the formation and growth of tumors, composed of tumor cells, immune cells, the vascular system, and other components [9, 10]. Abnormal vascular networks are among the important hallmarks of cancer. Tumor vessels not only provide necessary oxygen and nutrients for tumor tissues but also serve as the first barrier for immune cells to enter into tumor tissues, playing an instrumental role in immune regulation [11–13]. Currently, the expression of immunosuppressive molecules such as PD-L1 in the tumor microenvironment is one of the hot topics of research. Most attention is focused on the relationship between PD-L1 expression on tumor cells, its immunosuppressive effects, and the prognosis of patients. However, the expression status and mechanism of PD-L1 on tumor VECs of NPC, as well as its role in the tumor microenvironment, have yet to be explored.

The present study aims to determine the potential mechanism underlying the upregulation of PD-L1 expression on tumor VECs and its prognostic value in NPC. These findings can provide novel insights into identifying new prognostic markers for NPC and reversing the tumor immune escape mechanism.

## Results

### Upregulation of PD-L1 expression on tumor VECs of NPC

Although it has been reported that VECs can express PD-L1, little is known about PD-L1 expression on tumor VECs in NPC. Considering whether EBV could have an effect on the expression of PD-L1 on VECs, we first treated HUVECs with supernatants from EBV-positive and EBV-negative tumor cell lines (HK1-EBV, HK1, CNE2-EBV, CNE2, AGS-EBV, AGS). Western blot results indicated that compared with control-HUVECs, HUVECs treated with supernatants had higher PD-L1 expression, regardless of whether EBV was present or not (Supplementary Fig. 1). The result suggested that the upregulation of PD-L1 expression was not due to EBV. Given the high correlation between NPC and EBV, and considering that the vast majority of patients were clinically infected with EBV, we finally selected EBV-positive cell lines of NPC (C666-1 and HK1-EBV) for subsequent experiments.

**Fig. 1:**
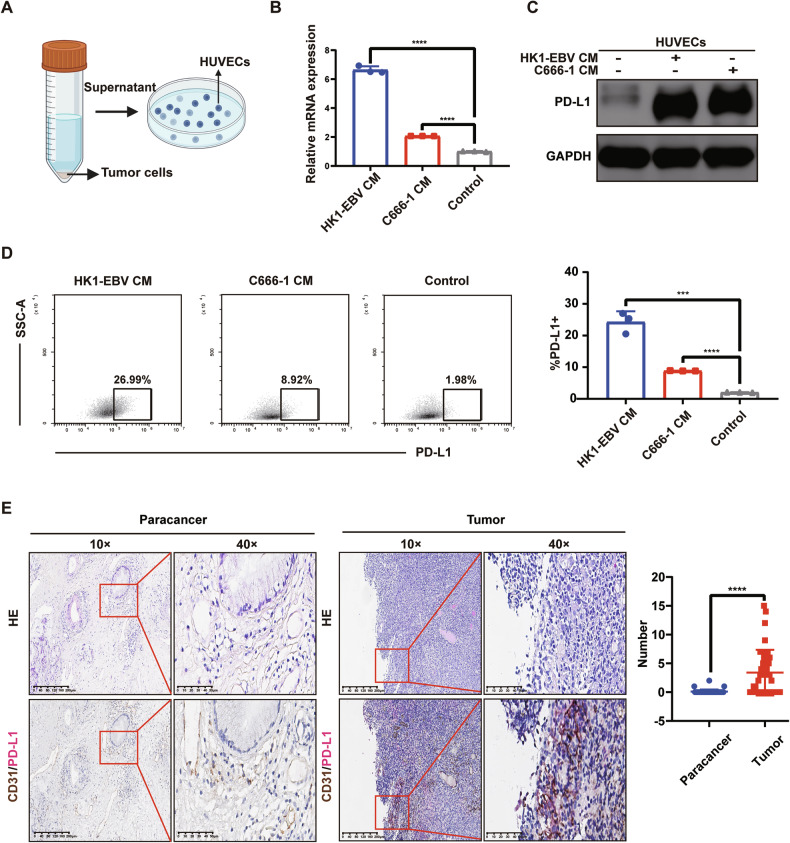
Upregulation of PD-L1 expression on tumor VECs of NPC. **A** Schematic representation of HUVECs treated with the supernatant from tumor cells (CM) of NPC. **B** After treatment with the supernatant of NPC for 48 h, qRT-PCR examined the PD-L1 mRNA in HUVECs of the supernatant-treated and untreated groups. Data are mean ± SD, *n* = 3, two-tailed Student’s *t*-test. **C** After treatment with the supernatant of NPC for 48 h, Western blot analysis of the expression of PD-L1 in HUVECs of the supernatant-treated group and the untreated group. **D** After treatment with the supernatant of NPC for 48 h, flow cytometry analysis of the expression of PD-L1 in HUVECs of the supernatant-treated group and the untreated group. Data are mean ± SD, *n* = 3, two-tailed Student’s *t*-test. **E** Double-staining immunohistochemistry specimens from 8 NPC patients were analyzed, revealing that PD-L1 expression on VECs was significantly higher in tumor tissues compared to paracancerous tissues. (Left) Typical images of double staining immunohistochemistry for CD31/PD-L1. (Right) A box plot. The number of CD31/PD-L1 co-localization was calculated respectively by randomly selecting 5 visual fields in tumor and paracancerous tissues of each patient’s section. Data are mean ± SD, *n* = 8, two-tailed t-test, ***P < 0.001, ****P < 0.0001.

We handled HUVECs with supernatants from the two aforementioned tumor cell lines for 48 h (Fig. 1A). We examined the transcription level of PD-L1 in HUVECs of the supernatant-treated and untreated groups by qRT-PCR. The results showed that PD-L1 mRNA levels were significantly elevated in HUVECs of the supernatant-treated group (*P* < 0.0001) (Fig. 1B). Next, we assessed the expression of PD-L1 in HUVECs of the supernatant-treated group and the untreated group by WB and flow cytometry. The results indicated that PD-L1 expression was significantly upregulated in HUVECs from the supernatant-treated group (Fig. 1C, D).

We conducted double-staining immunohistochemistry on tissue sections from 8 NPC patients and found that PD-L1 expression on VECs was significantly elevated in tumor tissues compared to paracancerous tissues (*P* < 0.0001) (Fig. 1E).

### PD-L1 expression on tumor VECs signed poorer prognosis of NPC patients

**Fig. 2:**
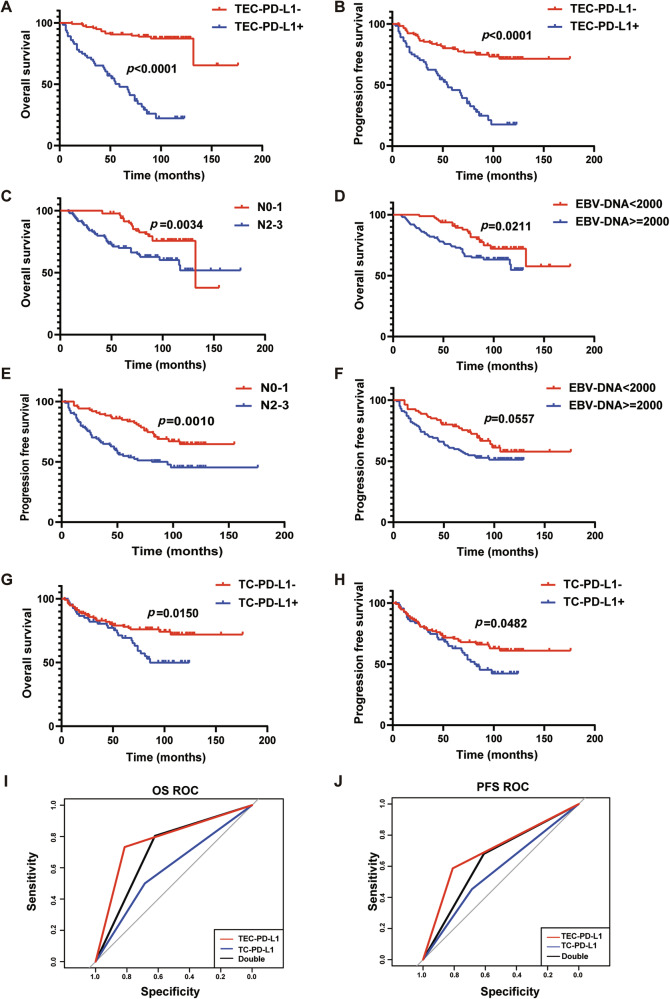
PD-L1 expression on tumor VECs signed poorer prognosis of NPC patients. **A**, **B** Kaplan–Meier survival curves of OS (**A**) and PFS (**B**) of patients with NPC exhibiting negative (red) or positive (blue) tumor VEC-PD-L1 (TEC-PD-L1). *n* = 180, Log-rank test. **C**, **E** Kaplan–Meier survival curves of OS (**C**) and PFS (**E**) of patients with NPC exhibiting N0-1 (red) or N2-3 (blue). *n* = 180, Log-rank test. **D**, **F** Kaplan–Meier survival curves of OS (**D**) and PFS (**F**) of patients with NPC exhibiting EBV-DNA < 2000 (red) or EBV-DNA > = 2000 (blue). *n* = 180, Log-rank test. **G**, **H** Kaplan–Meier survival curves of OS (**G**) and PFS (**H**) of patients with NPC exhibiting negative (red) or positive (blue) tumor cell-PD-L1 (TC-PD-L1). *n* = 180, Log-rank test. **I**, **J** ROC curves of TEC-PD-L1 (red), TC-PD-L1 (blue), and Double (black) predicting OS (**I**) and PFS (**J**) of NPC patients. *n* = 180, Log-rank test.

To evaluate the prognostic value of PD-L1 expressed on tumor VECs (tumor-VECs-PD-L1, TEC-PD-L1) in NPC patients, we analyzed the relationship between TEC-PD-L1 in 180 NPC tissue sections from Sun Yat-sen University Cancer Center and patients’ OS and PFS. The results showed that TEC-PD-L1-positive patients had worse outcomes in both OS and PFS (Fig. 2A, B). Moreover, univariate and multivariate Cox regression analyses showed that TEC-PD-L1 was an independent prognostic factor affecting OS and PFS in NPC patients (Table 1). Additionally, plasma EBV-DNA levels and N-stage were identified as independent prognostic factors for OS and PFS. We evaluated the effects of N-stage, plasma EBV-DNA on OS and PFS of NPC patients using Kaplan-Meier survival curves. The results suggested that patients with higher plasma EBV-DNA copy numbers had worse OS and PFS, and those with more advanced lymph node metastasis also exhibited worse OS and PFS (Fig. 2C–F).

**Table 1:** Univariate and multivariate analyses of factors associated with overall survival and progression-free survival in patients with nasopharyngeal carcinoma.

Variable	Overall survival				Progression-free survival			
	Univariate cox		Multivariate cox		Univariate cox		Multivariate cox	
	*P*-value	HR (95% CI)	*P*-value	HR (95% CI)	*P*-value	HR (95% CI)	*P*-value	HR (95% CI)
Age								
<50		1				1		
> =50	0.534	1.188 (0.690–2.046)			0.999	1.000 (0.628–1.591)		
Gender								
Female		1				1		
Male	0.751	0.908 (0.502–1.645)			0.464	0.830 (0.505–1.365)		
PD-L1 expression in Tumor vascular endothelial cells								
Negative		1		1		1		1
Positive	<**0.001**	8.300 (4.539–15.177)	**<0.001**	8.700 (4.747–15.943)	<**0.001**	4.130 (2.579-6.612)	**<0.001**	4.216 (2.624-6.776)
CD31								
Negative		1				1		
Positive	0.905	1.064 (0.384–2.951)			0.827	1.107 (0.446–2.743)		
T classification								
I-II		1				1		
III-IV	0.214	1.794 (0.713–4.514)			0.217	1.634 (0.750–3.558)		
N classification								
0-1		1		1		1		1
2-3	**0.006**	2.188 (1.253–3.818)	**0.040**	1.799 (1.026–3.154)	**0.001**	2.163 (1.348–3.471)	**0.014**	1.824 (1.127–2.951)
EBV-DNA								
<2000		1		1		1		1
> =2000	**0.030**	1.887 (1.062–3.353)	**0.010**	2.144 (1.198–3.836)	0.059	1.576 (0.983–2.527)	**0.049**	1.622 (1.000–2.631)
Treatment								
CCRT		1				1		
CCRT + IC/AC	0.399	1.307 (0.701–2.435)			0.582	1.159 (0.684–1.964)		
Radiotherapy	0.454	1.443 (0.553–3.767)			0.136	1.781 (0.833–3.807)		
Targeted therapy								
NO		1				1		
YES	0.698	0.898 (0.523–1.544)			0.731	0.922 (0.582–1.463)		

*CCRT*, concurrent chemoradiotherapy, *IC*, induction chemotherapy, *AC*, adjuvant chemotherapy. Bold values indicate significance at *P* < 0.05.

According to the literature, NPC patients with high levels of PD-L1 expression on tumor cells (TC/TPS ≥ 10%) may have a poorer prognosis [14]. Therefore, we analyzed the relationship between tumor cell PD-L1 (TC-PD-L1) and the prognosis of NPC patients. Kaplan-Meier results showed that patients with TC-PD-L1+ had worse OS than those with TC-PD-L1-, and the same was true for PFS (Fig. 2G, H).

To further compare the predictive performance of classification models, the receiver operating characteristics (ROC) curve and the area under the curve (AUC) were generated. According to the ROC curve, the AUC was greatest in the TEC-PD-L1 group compared to the TC-PD-L1 and Dual-scoring groups, for both OS and PFS (Fig. 2I, J and Supplementary Table 1). The above results suggested that the TEC-PD-L1 scoring system has superior predictive accuracy for survival outcomes.

### Upregulation of TEC-PD-L1 suppressed immune function and proliferation of T cells

**Fig. 3:**
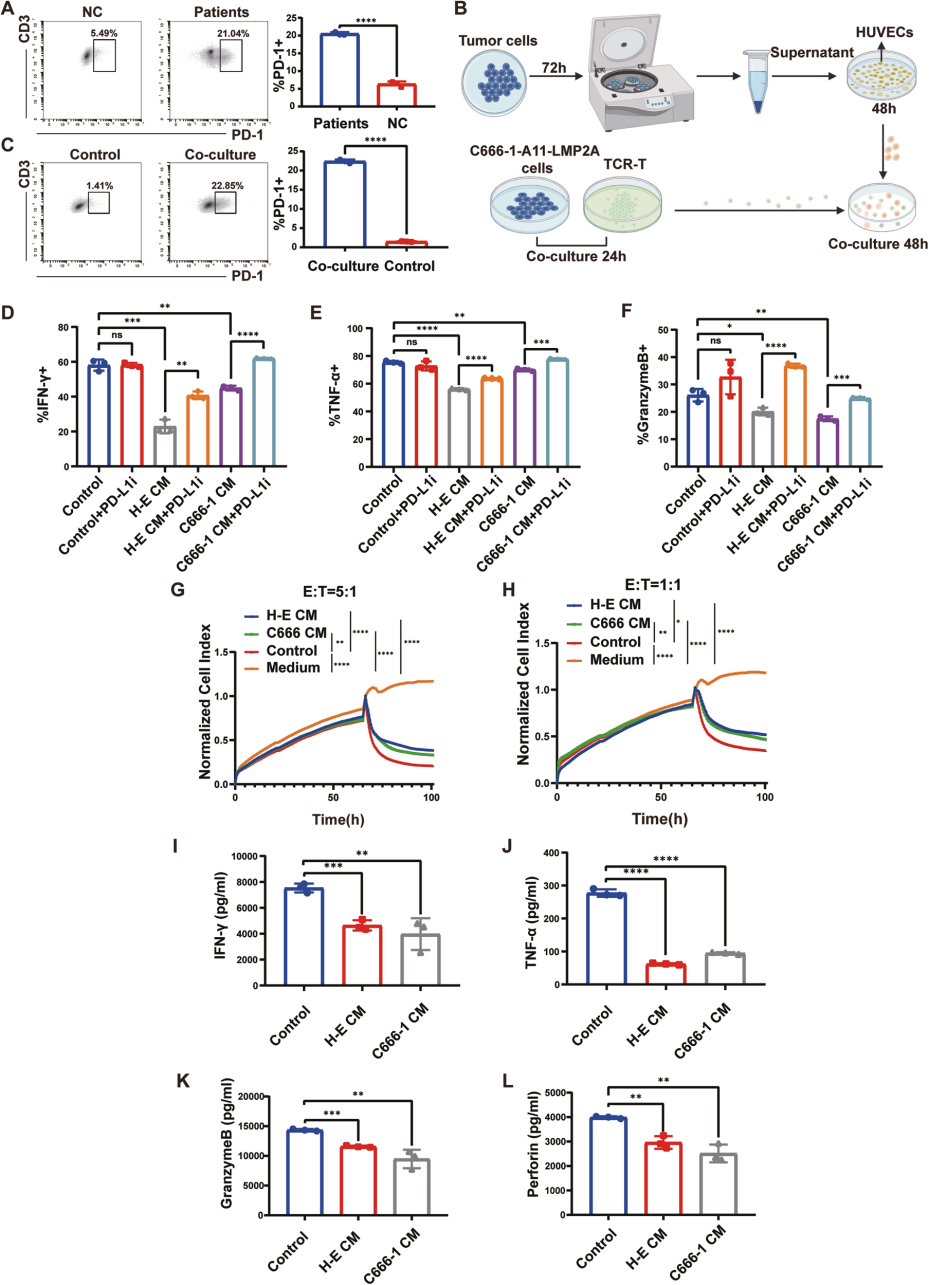
Upregulation of TEC-PD-L1 suppressed immune function and proliferation of T cells. **A** Expression of PD-1 on peripheral blood T cells from healthy individuals and patients with NPC. **B** Working model illustrating the co-culture system. **C** Expression of PD-1 on control TCR-T and co-culture TCR-T. **D**, **E**, **F** Statistical plots of the secretion levels of IFN-γ, TNF-α, and Granzyme B in TCR-T cells detected by flow cytometry after different treatments. *Control* indicates TCR-T co-incubated with control HUVECs, *H-E* indicates TCR-T co-incubated with HK1-EBV CM-treated HUVECs, *C666-1* indicates TCR-T co-incubated with C666-1 CM-treated HUVECs, and *PD-L1i* indicates incorporation of PD-L1 inhibitor. **G**, **H** At killing efficacy target ratios (E:T) of 5:1 (**G**) and 1:1 (**H**), CM-treated HUVECs resulted in a decrease in the antitumor capacity of TCR-T. **I**, **J**, **K**, **L** Secretion of IFN-γ, TNF-α, Granzyme B and Perforin in the supernatants of different co-culture systems. *Control* indicates supernatants from the co-culture system of control HUVECs with TCR-T, *H-E CM* indicates supernatants from the co-culture system of HUVECs treated with HK1-EBV supernatant with TCR-T, and *C666-1 CM* indicates supernatants from the co-culture system of HUVECs treated with C666-1 supernatant with TCR-T. Data are mean ± SD, *P < 0.05, **P < 0.01, ***P < 0.001, ****P < 0.0001, ns, no significance, two-tailed *t*-test.

Since the PD-1/PD-L1 signaling pathway plays a crucial role in mediating tumor immunosuppression, we speculated whether T cells were suppressed from performing their normal functions by PD-L1 expressed on tumor VECs before reaching tumor cells. Given that PD-L1 typically binds to PD-1 expressed on activated T cells, transmitting immunosuppressive signals, we first examined whether circulating T cells from patients expressed PD-1. Using flow cytometry, we compared PD-1 expression on peripheral blood T cells between healthy individuals and NPC patients, finding that PD-1 expression was significantly elevated in peripheral blood T cells from NPC patients compared to healthy individuals (Fig. 3A).

To simulate this phenomenon in vivo, we aimed to adjust PD-1 expression on T cells to levels comparable to in vivo conditions in cellular experiments. Since activation of the T cell receptor (TCR) can upregulate PD-1 expression, we used MHC-matched pairs of TCR-T cells and the C666-1-A11-LMP2A cell line. The TCR-T cells recognized the antigenic peptide LMP2A on C666-1-A11-LMP2A and were MHC-compatible with these cells (Supplementary Fig. 2). We co-incubated the constructed TCR-T with C666-1-A11-LMP2A to up-regulate PD-1 expression on TCR-T. In addition, we used C666-1 cells as well as supernatants from NPC cells (C666-1 and HK1-EBV) for co-incubation with TCR-T cells, respectively, and then detected PD-1 expression on the TCR-T cells. The most significant increase in PD-1 expression on TCR-T cells was observed after co-incubation with C666-1-A11-LMP2A cells compared to the other treatments. Furthermore, a 24-h co-incubation period resulted in the most pronounced elevation of PD-1 on TCR-T cells, similar to the expression of PD-1 on T cells in the peripheral-blood of NPC patients (Supplementary Fig. 3). Therefore, we selected the culture conditions of ‘24 h’ and ‘co-incubation with C666-1-A11-LMP2A’ to increase PD-1 expression on TCR-T cells for subsequent experiments.

Next, we conducted co-culture experiments following the flowchart in Fig. 3B. Firstly, we assessed the expression of PD-1 on co-cultured TCR-T cells by flow cytometry (Fig. 3C). Subsequently, we co-cultured the TCR-T cells, which exhibited PD-1 expression levels comparable to those of T cells in NPC patients, with supernatant-treated HUVECs for 48 h. Finally, we examined the levels of IFN-γ, TNF-α, and GranzymeB in TCR-T by flow cytometry. The results showed that after co-incubation with supernatant-treated HUVECs, secretion of IFN-γ, TNF-α, and GranzymeB by TCR-T cells was significantly reduced (Fig. 3D–F and Supplementary Fig. 4). Moreover, the secretion of functional molecules by TCR-T cells was restored upon the addition of a PD-L1 inhibitor (1 μg/ml) to the co-culture of TCR-T cells and HUVECs (Fig. 3D–F and Supplementary Fig. 4). This suggested that the reduced secretion of functional molecules by T cells was attributable to the interaction between PD-1 on TCR-T cells and PD-L1 on CM-HUVECs.

To better mimic the conditions in NPC patients, we used peripheral blood T cells from NPC patients and healthy human peripheral blood T cells coincubated with supernatant-treated HUVECs 48 h, respectively. Next, we detected the secretion of T-cell killer molecules (TNF-α, IFN-γ, Granzyme B). The results suggested that after co-culture, peripheral blood T cells from NPC patients secreted significantly less TNF-α, IFN-γ, and Granzyme B compared with healthy human peripheral blood T cells (Supplementary Fig. 5).

We performed cytotoxicity assays using the xCELLigence Real-Time Cell Analyzer-Multiple Plate system (Agilent Technologies) at 5:1 and 1:1 effector to target (E: T) ratios for 100 consecutive hours to assess the anti-tumor advantage of TCR-T in different treatment groups. We found that TCR-T co-incubated with control-HUVECs was more capable of killing tumors (Fig. 3G, H). We collected supernatants from each group of the co-culture system for ELISA assays, and the results suggested that TCR-T co-incubated with CM-HUVECs secreted fewer functional cytokines (Fig. 3I–L).

Finally, we evaluated the effect of tumor VECs on the proliferative capacity of T cells and found that HUVECs with high PD-L1 expression inhibited the proliferative capacity of T cells (Supplementary Fig. 6).

### Tumor VECs weakened the anti-tumor capacity of T cells in vivo

**Fig. 4:**
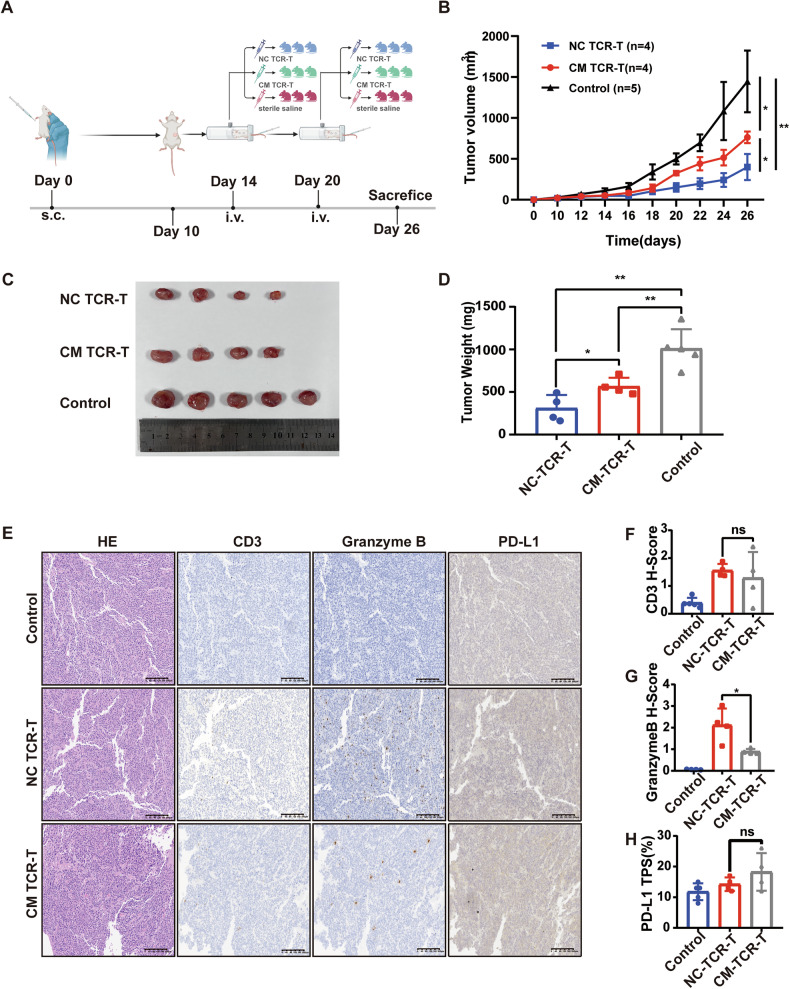
Tumor VECs weakened the anti-tumor capacity of T cells in vivo. **A** Schematic diagram of animal experiment. NCG mice were intravenously injected with 5 million C666-1-A11-LMP2A cells. TCR-T co-incubated with control-HUVECs (NC-TCR-T), and TCR-T co-incubated with tumor supernatant-treated HUVECs (CM-TCR-T) were injected into mice by tail vein at a dose of 1*10^7^ on days 14 and 20, and a separate group of mice was infused back with sterile saline as a control group. **B** Tumor growth curve in different groups. Mean ± SD, two-way ANOVA**. C** At 26 days after tumor inoculation, tumors were isolated from mice in each group. **D** At day 26, after tumor inoculation, tumor weight was determined in each group. Data are mean ± SD, two-tailed *t*-test. **E** Representative images of hematoxylin and eosin (H&E) staining and IHC staining of CD3, Granzyme B, and PD-L1 in mice from each group. **F** Statistical plot of H score of CD3 in tumor slices of each group of mice. Data are mean ± SD, two-tailed t-test. **G** Statistical plot of H score of Granzyme B in tumor slices of each group of mice. Data are mean ± SD, two-tailed *t*-test. **H** Statistical plot of PD-L1 TPS in tumor slices of each group of mice. Data are mean ± SD, two-tailed t-test. *P < 0.05, **P < 0.01, ***P < 0.001, ****P < 0.0001, ns, no significance.

We subcutaneously inoculated NCG mice with C666-1-A11-LMP2A cells to establish xenograft tumors. TCR-T cells co-incubated with control-HUVECs (NC-TCR-T) and TCR-T cells co-incubated with tumor supernatant-treated HUVECs (CM-TCR-T) were injected into mice via tail vein at a dose of 1 × 10^7^ cells on days 14 and 20. A separate group of mice was infused with sterile saline as a control (Fig. 4A). The tumor growth curve indicated that the tumor growth rate in the CM-TCR-T group significantly exceeded that in the NC-TCR-T group (Fig. 4B). At the end of the animal experiment on day 26, the mice were euthanized to excise the tumors, and the tumor volume and weight in the CM-TCR-T group significantly surpassed those in the NC-TCR-T group (Fig. 4C, D).

To assess the number and function of T cells within mouse xenograft tumors, we carried out IHC staining of xenograft tumor tissue sections using human CD3 antibody and human Granzyme B antibody (Fig. 4E). The results demonstrated that there was no significant difference in CD3 staining between the NC-TCR-T group and the CM-TCR-T group (*P* > 0.05) (Fig. 4F). However, Granzyme B staining was significantly stronger in the NC-TCR-T group than in the CM-TCR-T group (*P* < 0.05) (Fig. 4G). In addition, we employed IHC to evaluate the expression of PD-L1 in tumor sections of mice within each group (Fig. 4E). The result indicated that there was no significant difference in PD-L1 TPS among the tumor sections of mice between NC-TCR-T and CM-TCR-T groups (*P* > 0.05) (Fig. 4H), which can exclude the effect of tumor cell PD-L1 on TCR-T. The above results indicated that CM-HUVECs attenuated the anti-tumor ability of TCR-T cells by inhibiting their function rather than by reducing their intratumoral infiltration.

### Tumor supernatants of NPC upregulated PD-L1 expression on HUVECs via crosstalk between NF-κB and STAT3 signaling pathways

**Fig. 5:**
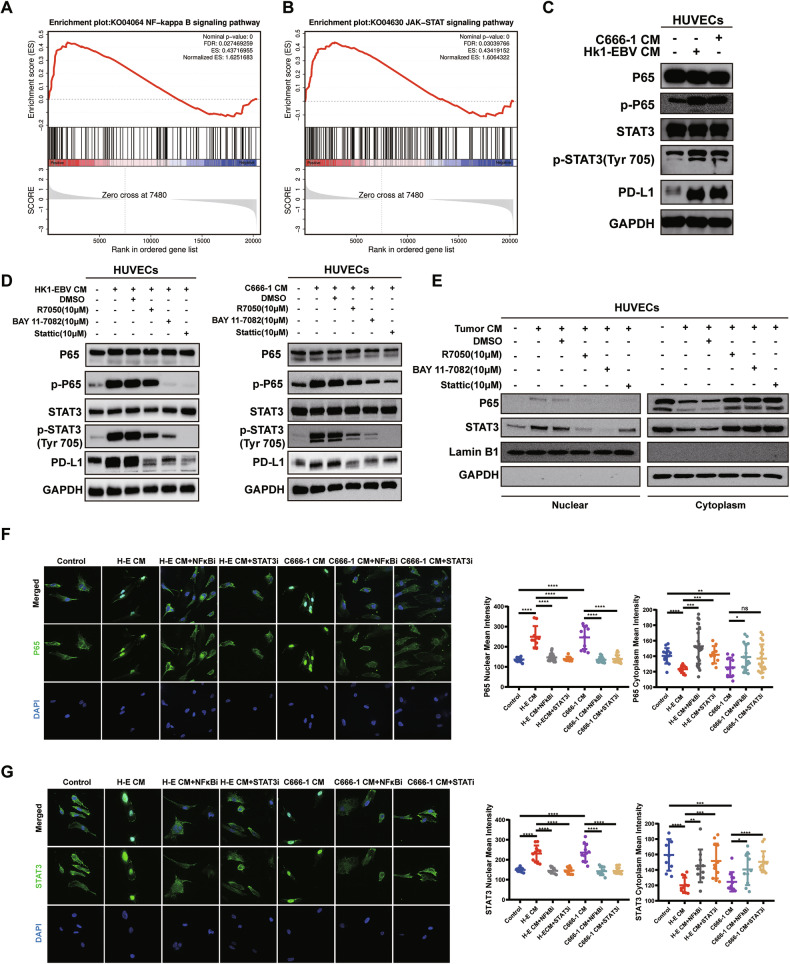
Tumor supernatants of NPC upregulated PD-L1 expression on HUVECs via crosstalk between NF-κB and STAT3 signaling pathways. **A**, **B** GSEA results indicated that the JAK-STAT and NF-κB pathways were significantly upregulated. **C** HUVECs were pretreated with or without tumor CM of NPC. The expression of phospho-P65, total P65, phospho-STAT3, total STAT3, and PD-L1 was determined by WB. **D** HUVECs were pretreated with or without NF-κB or STAT3 signaling pathway inhibitors for 1 h, followed by co-culture with or without tumor CM of NPC. The expression of phospho-P65, total P65, phospho-STAT3, total STAT3, and PD-L1 was determined by WB. **E** HUVECs were pretreated with or without NF-κB or STAT3 signaling pathway inhibitors for 1 h, followed by co-culture with or without tumor CM of NPC. WB detection of P65 and STAT3 in the nuclear and cytoplasm, respectively. **F** Immunofluorescence assay to determine the effect of STAT3 pathway inhibitor (STATi, 10 μM) and NF-κB pathway inhibitor (NF-κBi, 10 μM) and supernatants of NPC tumor cell (H-E, C666-1) on P65 nuclear translocation in HUVECs. (Left) representative immunofluorescence images of P65 staining. Magnification: 1000×. (Right) The mean density values of P65 levels in the cytoplasm and nucleus of HUVECs in each group. Data are mean ± SD, two-tailed *t*-test. **G** Immunofluorescence assay to determine the effect of STAT3 pathway inhibitor (STATi, 10 μM) and NF-κB pathway inhibitor (NF-κBi, 10 μM) and supernatants of NPC tumor cell (H–E, C666-1) on STAT3 nuclear translocation in HUVECs. (Left) representative immunofluorescence images of STAT3 staining. Magnification: 1000×. (Right) The mean density values of STAT3 levels in the cytoplasm and nucleus of HUVECs in each group. Data are mean ± SD, two-tailed t-test. *P < 0.05, **P < 0.01, ***P < 0.001, ****P < 0.0001, ns, no significance.

To explore the potential mechanisms by which tumor supernatants of NPC upregulate PD-L1 expression on HUVECs, we subjected NPC tumor supernatant-treated and untreated HUVECs to transcriptome sequencing. GESA enrichment analysis showed significant upregulation of both the NF-κB signaling pathway and the JAK-STAT signaling pathway in HUVECs treated with tumor supernatants (Fig. 5A, B). Western blot results suggested upregulation of p-P65, p-STAT3, and PD-L1 in supernatant-treated HUVECs. (Fig. 5C).

HUVECs were pretreated with NF-κB signaling pathway inhibitors R7050 (10 μM) [15] and BAY 11-7082 (10 μM) [16], as well as the STAT3 inhibitor Stattic (10 μM) [17], for 1 h before the addition of NPC tumor cell supernatants. Western blot results suggested that the NF-κB pathway inhibitors could downregulate p-P65, p-STAT3, and PD-L1 on HUVECs treated with tumor supernatants (Fig. 5D). Similarly, addition of the STAT3 inhibitor led to downregulation of p-P65, p-STAT3, and PD-L1 in HUVECs treated with tumor supernatants (Fig. 5D). In summary, NPC cell supernatants may upregulate PD-L1 expression on HUVECs by inducing phosphorylation of P65 and STAT3, highlighting mutual crosstalk between the NF-κB and STAT3 signaling pathways.

Since both P65 and STAT3 are common transcription factors, phosphorylated P65 and STAT3 can form homo- or heterodimers that translocate into the nucleus to initiate transcription of target genes. Hence, we investigated whether STAT3 and P65 enter the nucleus to play a transcriptional role. Western blot and cellular immunofluorescence experiments revealed that NPC cell supernatants induced the nuclear translocation of P65 and STAT3 in HUVECs, and the addition of NF-κB inhibitor and STAT3 inhibitor reversed this nuclear translocation phenomenon (Fig. 5E–G).

## Discussion

Tumor vasculature is an integral component of the tumor microenvironment, playing a crucial role in facilitating immune cell delivery to tumor tissues. Studies have shown that tumor angiogenesis and immunosuppression go hand in hand [18–21]. In addition, tumor vasculature is structurally and functionally distinct from normal vasculature [22, 23]. Given the current focus on immunosuppressive molecules within the tumor microenvironment, we speculated whether T cells might be hindered in their anti-tumor capacity by such molecules, such as PD-L1, expressed on tumor VECs during their initial entry into the tumor vasculature. In this study, we observed upregulation of PD-L1 expression on tumor VECs in NPC at both cellular and tissue levels. Previous studies have reported similar findings in lung adenocarcinoma, renal cancer, and colon cancer [24], consistent with our results.

It has been proposed that tumor VECs establish a substantial barrier limiting T-cell infiltration, termed the tumor vascular endothelial barrier [20]. Above all, the vascular endothelium serves as a passive physical barrier for the infiltration of immune cells, and relevant studies have focused on the interactions between the vascular endothelium, responsible for T-cell transport, and T-cell adhesion [20]. In addition to the barrier role, tumor VECs also regulate the immunoreactivity of T cells. Echoing the findings of several studies [25, 26], our results similarly showed that in NPC, tumor VECs facilitated tumor immune escape by upregulating the immunosuppressive ligand PD-L1. This study revealed for the first time that tumor VECs exhibit plasticity in response to signals from the tumor microenvironment of NPC, transforming VECs into unfavorable immunosuppressive mediators.

PD-L1 detected by immunohistochemistry has become the most widely validated, used, and accepted biomarker for screening patients for immune checkpoint inhibitor therapy [27]. In NPC, the Captain study and Polaris-02 study have shown that patients with high PD-L1 expression on tumor cells have better therapeutic efficacy of PD-1 inhibitors compared to patients with low PD-L1 expression [14, 28]. Nevertheless, the value of PD-L1 for predicting the prognosis of NPC patients treated with chemoradiotherapy is still debatable. In NPC, several studies have claimed that high expression of PD-L1 on tumor cells signifies a better prognosis [29–31], while others have concluded the opposite, stating that high expression of PD-L1 on tumor cells is associated with poorer clinical outcomes in patients [32–35]. Moreover, some studies have suggested that the expression of PD-L1 on tumor cells is not relevant to survival in NPC [36, 37]. In our study, high expression of PD-L1 on tumor cells predicted worse clinical outcomes in patients. Regarding the differences in the results of different studies, we believe that, firstly, the defining points of high and low PD-L1 expression on tumor cells may be different. Secondly, there may be variations in the interpretation of PD-L1 positive results by different pathologists. In addition, differences in detection antibodies and platforms may also cause different results.

Studies on the relationship between PD-L1 expression on tumor vasculature, an important component of the tumor microenvironment, and prognosis are extremely rare, especially in NPC, where no studies have been reported. From what we found, TEC-PD-L1 was an independent risk factor affecting OS and PFS in NPC patients, and patients with positive TEC-PD-L1 were worse in both OS and PFS. A previous study reported that in lung adenocarcinoma, high expression of TEC-PD-L1 predicted worse OS and PFS in patients [24], which was consistent with our findings. Next, we compared the value of TC-PD-L1, TEC-PD-L1, and the combination of the two for assessing the prognosis of NPC patients. The results of the ROC curves showed that the AUC of the TEC-PD-L1 group was the largest in both OS and PFS, demonstrating that TEC-PD-L1 had the best prognostic predictive value for NPC patients. Based on the above, we were the pioneer in proposing that a new prognostic indicator, TEC-PD-L1, may be an essential biomarker for the prognosis of NPC patients. Tumor vasculature is the first step for T cells to enter the neoplasm, so PD-L1 expressed on the first line of defense is the foremost factor in diminishing the anti-tumor function of T cells, which may indicate a poorer prognosis for patients.

For the mechanism of PD-L1 upregulation, most previous studies have focused on PD-L1 expressed by tumor cells. However, little research has been done on the mechanism of PD-L1 upregulation in tumor VECs. Prior literature reported that hypoxia could induce HUVECs to express HIF-α and upregulate PD-L1 expression [38]. In this study, we found for the first time that the NF-κB and STAT3 signaling pathways may play a role in upregulating PD-L1 expression on tumor VECs in NPC. This finding provides a new perspective for subsequent studies on whether combining inhibitors of these pathways with PD-1/PD-L1 inhibitors could increase treatment efficacy for NPC.

Normalizing the components of the tumor microenvironment inhibits tumor progression and improves therapeutic outcomes. From this perspective, improving the tumor vasculature, an important component of the tumor microenvironment may also be crucial for inhibiting tumor immune escape. Current therapies targeting the tumor vasculature, such as anti-angiogenic drugs, can both counteract angiogenesis and normalize abnormal tumor vasculature [39]. Thus, we speculated whether anti-angiogenic drugs could downregulate the expression of PD-L1 on abnormal tumor VECs. Research has shown that the anti-angiogenic drug anlotinib could inhibit the upregulation of HUVECs-PD-L1 induced by lung cancer cell supernatants [24]. In NPC, the small molecule anti-angiogenic drug apatinib is a promising therapeutic agent. Exploring whether apatinib can inhibit the upregulation of PD-L1 expression on HUVECs caused by NPC cell supernatants is our next task. Combining immune checkpoint inhibitors with anti-angiogenic drugs is a promising topic in tumor therapy. Currently, the PD-1 inhibitor Camrelizumab combined with apatinib has long-lasting antitumor activity in patients with recurrent or metastatic NPC [40]. Our future studies may provide partial insights into the mechanism of potentiation of PD-1/PD-L1 inhibitors in combination with anti-angiogenic drugs.

## Conclusions

**Fig. 6:**
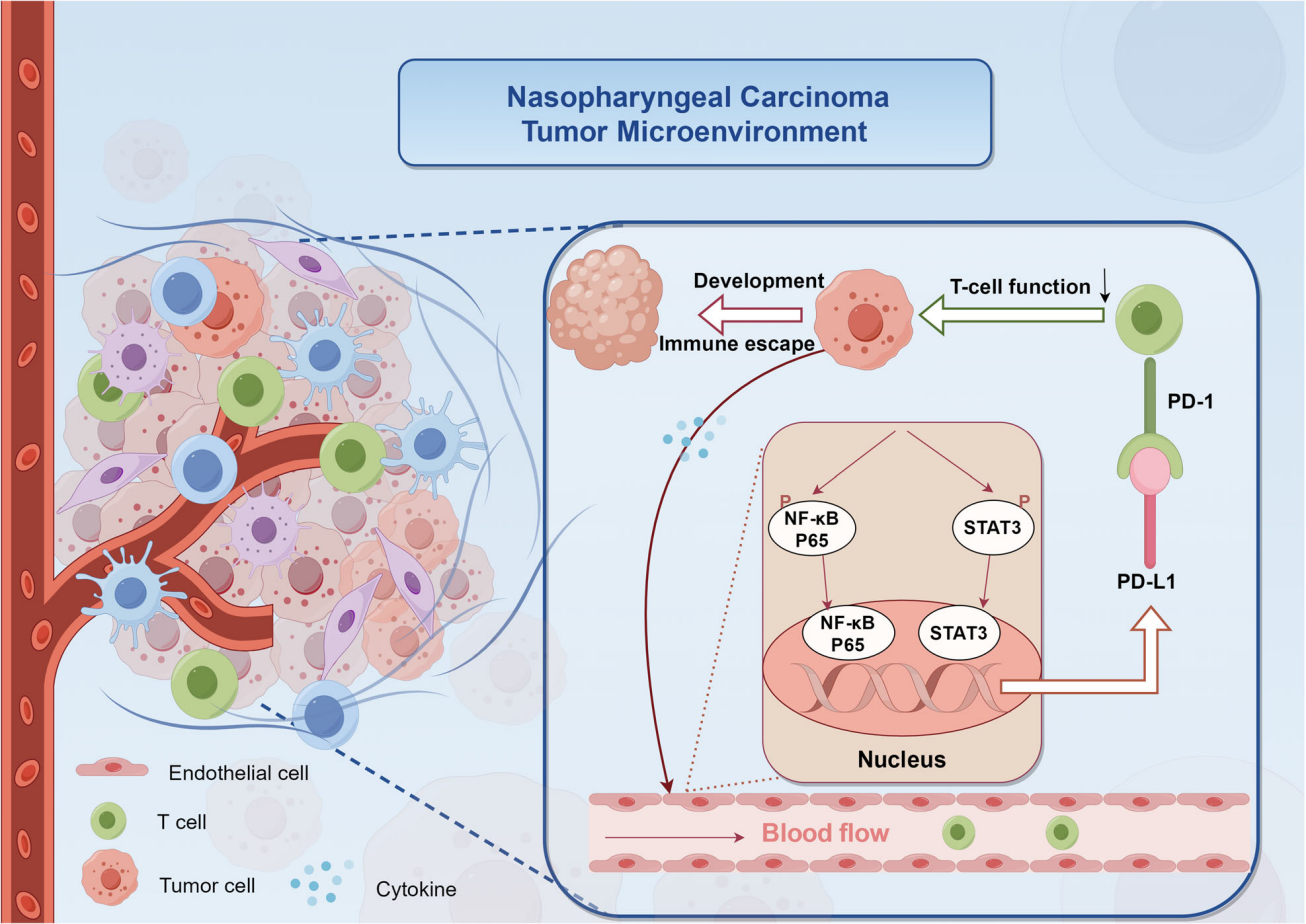
Schematic depicting the interaction between VECs and T cells and tumor cells in the tumor microenvironment of NPC. In the tumor microenvironment of nasopharyngeal carcinoma, endothelial cells exhibit activation of both NF-κB and JAK-STAT signaling pathways in response to "tumor cell-derived signals". This activation triggers phosphorylation and nuclear translocation of P65 and STAT3 in endothelial cells, resulting in the upregulation of PD-L1 expression. The interaction between upregulated PD-L1 and PD-1 on circulating T cells within the vasculature initiates immunosuppressive signaling, consequently impairing T cell-mediated cytotoxicity. This cascade of events ultimately facilitates immune evasion and tumor progression.

In summary, this study was the first to explore the role and mechanism of tumor VECs in tumor immune escape in NPC. We elucidated that overexpression of PD-L1 on tumor VECs in NPC was closely associated with poor prognosis. In addition, NPC tumor supernatants may promote the up-regulation of PD-L1 expressed on VECs by activating the NF-κB and STAT3 signaling pathways. Blocking these pathways may inhibit T cell inactivation and tumor immune escape induced by the upregulation of PD-L1 on tumor VECs (Fig. 6). Our study provided new insights for finding new prognostic markers and inhibiting immune escape in NPC.

## Materials and methods

### Cell lines and cell culture

We purchased human umbilical vein endothelial cells (HUVECs) from iCell Bioscience Inc. (Shanghai, China). The endothelial cell culture medium (ECM) (ScienCell, LA, USA), containing endothelial cell growth supplement (ECGS), 10% fetal bovine serum (FBS) (Gibco, NY, USA), and 1% penicillin/streptomycin (Invitrogen, Carlsbad, USA), was used for cell culture. Sun Yat-sen University Cancer Center (Guangzhou, China) provided the human NPC cell lines HK1-EBV, HK1, C666-1, CNE2, and CNE2-EBV, as well as the gastric cancer cell lines AGS and AGS-EBV. During the culturing of C666-1, Dulbecco’s modified Eagle’s medium (DMEM) (Gibco, NY, USA) supplemented with 10% FBS was used. The remaining cell lines were cultured in Roswell Park Memorial Institute (RPMI) 1640 medium (Gibco, NY, USA) supplemented with 10% FBS. TCR-T cells compatible with C666-1-A11-LMP2A cells were offered by TCRCure Biological Technology Company (Guangzhou, China). DMEM supplemented with 10% FBS was used for C666-1-A11-LMP2A cells, and X-Vivo medium (LONZA, Guangzhou, China) + 1000IU/ml IL2 (Beijing Sihuan Biopharmaceutical Co., Ltd, Beijing, China) was used for TCR-T cells. All cells were cultured in a humidified incubator at a constant temperature of 37 °C with 5% CO2.

### Patients and samples

We collected paraffin-embedded tumor specimens for performing immunohistochemical analyses from 180 patients who were initially diagnosed pathologically with non-metastatic NPC at Sun Yat-sen University Cancer Center between 2008 and 2019. Of the NPC patients’ tissues used in this study, seven patients were treated by chemo/radiotherapy and the rest were naïve.

### Co-culture experiment

One day in advance, HUVECs were inoculated in cell culture flasks or dishes at a density of about 30-40%. The next day, the supernatant was changed by adding NPC tumor cell supernatants and ECM + 10% FBS at a 1:1 ratio and incubated for 48 h. Afterwards, the expression of PD-L1 in HUVECs was detected or followed up by subsequent experiments.

We co-incubated TCR-T with C666-1-A11-LMP2A at a ratio of 5:1 to upregulate PD-1 expression on TCR-T. Subsequently, the co-incubated TCR-T cells were co-cultured with supernatant-treated and untreated HUVECs for 48 h. Flow cytometry was then used to detect the levels of cytokines in TCR-T or collect TCR-T cells for subsequent experiments. We conducted independent experiments using TCR-T constructed from 3 patients.

### Immunohistochemistry (IHC)

A 2-h incubation at 65 °C was followed by deparaffinization and rehydration, citrate-mediated high-temperature antigen retrieval, treatment with 3% H2O2, blocking in goat serum, and incubation with primary antibodies at 4 °C overnight. Secondary antibodies were then incubated with the samples. Double staining immunohistochemistry was performed according to the kit instructions (#DS-0003, #DS-0004, ZS, Beijing, China). We scanned the slides using a digital pathology slide scanner (KFBIO, Ningbo, China) to achieve overall scanning results. Slides were assessed by 2 independent pathologists who were blind of the patients’ information. TEC-PD-L1 staining was defined by the following criteria: positive (presence of co-localization of CD31 and PD-L1 by double staining immunohistochemistry); negative (absence of co-localization of CD31 and PD-L1 by double staining immunohistochemistry). TC-PD-L1 staining was defined as follows: negative (<10% positive tumor cells); positive (≥10% positive tumor cells).

### Flow cytometry

Cells were processed as indicated, then collected and stained with the appropriate flow antibodies. Stained cells were analyzed using a cytoFLEX LX instrument with CytExpert software.

### Real-time quantitative polymerase chain reaction (qPCR)

Total RNA was isolated using an RNA Quick Purification Kit (Esscience, Shanghai, China). The Fast Reverse Transcription Kit (ESscience) was used to synthesize first-strand cDNA. Using LightCycler 480 (ROCHE), mRNA expression was measured. The sequences of the primers are as follows:

PD-L1-F: 5′-TGGCATTTGCTGAACGCATTT-3′,

PD-L1-R: 5′-TGCAGCCAGGTCTAATTGTTTT-3′;

GAPDH-F: 5′-GGAGCGAGATCCCTCCAAAAT-3′,

GAPDH-R: 5′-GGCTGTTGTCATACTTCTCATGG-3′.

### Western blot analysis

Cell samples were lysed in RIPA buffer containing phosphatase inhibitors (Sigma, Darmstadt, Germany). Proteins were tested using primary antibodies. Enhanced chemiluminescence (ECL) was used to visualize immunoreactive bands on the blots incubated with species-specific HRP-conjugated secondary antibodies.

### Cellular immunofluorescence

Three washes of cold PBS were performed, 4% paraformaldehyde was applied, PBS washed, and 1% Triton X-100 was applied for 10–15 min at room temperature. The next step involved blocking cells with goat serum for 30 min, washing them in PBS, and incubating them with primary antibodies for 1 h, thereafter incubating with Alexa Fluor 488 goat anti-mouse or goat anti-rabbit IgG (Invitrogen) for 1 h. Cells were then counterstained with DAPI (Sigma) and imaged with Nikon CSU-W1.

### Antibodies and chemicals

The antibodies used in this study were: PD-L1 (#13684 s, Cell Signaling Technology, Danvers, MA, USA 1:1000 for WB), P65 (#8242S, Cell Signaling Technology, 1:1000 for WB), p-P65 (#3033 T, Cell Signaling Technology, 1:1000 for WB), STAT3 (#9139 T, Cell Signaling Technology, 1:1000 for WB), p-STAT3 (#9145 T, Cell Signaling Technology, 1:2000 for WB), Lamin B1 (#12987-1-AP, Proteintech, IL, USA, 1:2000 for WB), GAPDH (#10494-1-AP, Proteintech, 1:1000 for WB), PD-L1 (#ab228462, Abcam, Cambridge, MA, USA, 1:100 for double staining IHC), CD31(#3528S, Cell Signaling Technology, 1:3200 for double staining IHC), GranzymeB (#46890S, Cell Signaling Technology, 1:200 for IHC), CD3 (#ZA-0503, ZS, for IHC), Fixable Viability Stain 780 (#565388, BD Biosciences, San Jose, CA, USA, for flow cytometry), PE/Cyanine7 anti-human CD3 (#300316, Biolegend, San Diego, CA, USA, clone: HIT3a, for flow cytometry), PE anti-human TNF-α (#502909, Biolegend, clone: MAb11, for flow cytometry), BV421 Mouse Anti-Human CD279 (#562516, BD Biosciences, clone EH12.1, for flow cytometry), APC anti-human IFN-γ (#502512, Biolegend, clone: 4S.B3, for flow cytometry), BV421 Mouse Anti-Human Granzyme B (#563389, BD Biosciences, clone: GB11, for flow cytometry), PE Mouse Anti-Human CD3 (#555333, BD Biosciences, clone: UCHT1, for flow cytometry), APC Mouse Anti-Human PD-L1 (#568315, BD Bioscience, clone: 29E.2A3, for flow cytometry), CellTrace™ CFSE (#C34570, Invitrogen, for flow cytometry), PE-Cy™7 Mouse anti-Ki-67 (#561283, BD Bioscience, clone: B56, for flow cytometry). R7050, BAY 11-7082, and Stattic were purchased from MedChem Express (MCE, Monmouth Junction, NJ, USA).

### ELISA

The concentrations of IFN-γ, TNF-α, GranzymeB, and Perforin in the supernatant of the purpose were detected according to the protocol of ELISA kit (Neobioscience and Dakewe Biotech Co., Ltd, Shenzhen, China).

### Tumor killing assays

Real-time cytotoxicity assays were conducted using the xCELLigence Real-Time Cell Analyser-Multiwell Plate System from Agilent Technologies (Santa Clara, CA, USA). In short, 5 × 10^4^ C666-1-A11-LMP2A cells were added to each well of a 16-well plate (#20220922). When the impedance rose to 0.8–1.2, the corresponding TCR-T cells with different E: T ratios were added. Biocompatible microelectrodes have a cell index value that represents changes in electrical impedance and can indicate the number of target cells surviving on their surface. The cell index was calculated every 15 min using the RTCA software. Each set of cell index data represents the average of 4 wells.

### Animal studies

Female NCG mice of 4-6 weeks were purchased from Guangdong GemPharmatech Co., Ltd (Foshan, China). The mice were randomly divided into three groups of five mice each, and each mouse was injected subcutaneously with 5 × 10^6^ tumor cells (C666-1). Since one mouse in each of the NC-TCR-T and CM-TCR-T groups had no subcutaneous tumor formation, four mice in each of these two groups were included in the subsequent experiments. On days 14 and 20, the transfusion of TCR-T cells was performed. Each mouse in the treatment group was infused with 1*10^7^ cells (CM-TCR-T or NC-TCR-T), and the control group was infused with saline. Tumor size was measured every two days and tumor volume was calculated as volume = (length × width^2^)/2. At the end of the experiment, tumors were stripped from euthanized mice, weighed, and embedded. Sections were subjected to IHC and hematoxylin-eosin (H&E) staining.

### Statistical analysis

GraphPad Prism 8.0.1 (GraphPad Software, Inc.), SPSS 20.0 (IBM Corp), and R version 4.2.1 were taken for statistical analyses. Student’s *t*-test, log-rank test, one-way or two-way ANOVA, as well as univariate and multivariate Cox regression analyses, were used for data analysis. All tests were conducted with a two-sided approach. Signifcant diferences were determined as: *P < 0.05, **P < 0.01, ***P < 0.001, and ****P < 0.0001.

## Supplementary information

Supplementary figures and tables

Supplementary material

## Data Availability

The datasets generated during and/or analyzed during the current study are available from the corresponding author upon reasonable request.
