# Supplementary material for: Tumor vascular endothelial cells promote immune escape by upregulating PD-L1 expression via crosstalk between NF-κB and STAT3 signaling pathways in nasopharyngeal carcinoma
Source: Cell Death Dis. 2025 Feb 25;16(1):129. doi: 10.1038/s41419-025-07444-z (PMC11861260; doi:10.1038/s41419-025-07444-z)
Supplement: Supplementary file 1 — Supplementary figures and tables [file 41419_2025_7444_MOESM1_ESM.pdf]

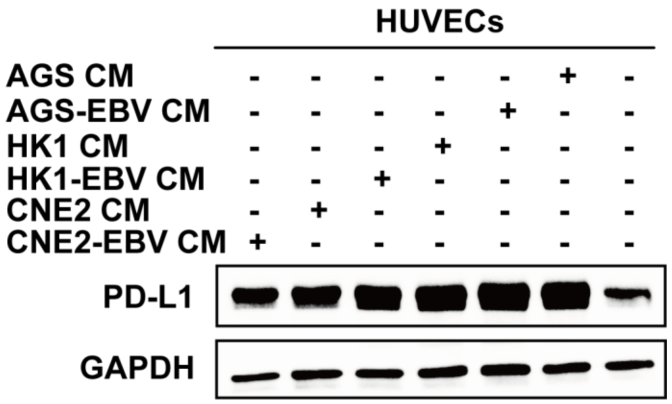

Supplementary Fig. 1. **PD-L1 expression in HUVECs treated with supernatants from EBV- and EBV+ tumor cells for 48h using WB detection.**

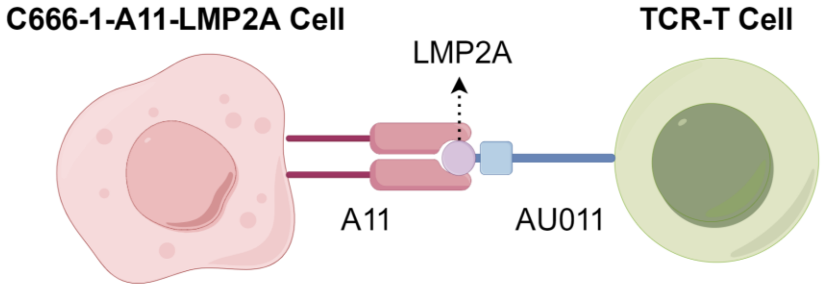

Supplementary Fig. 2. **Schematic diagram depicting TCR-T cells recognising C666-1-A11-LMP2A tumour cells.**

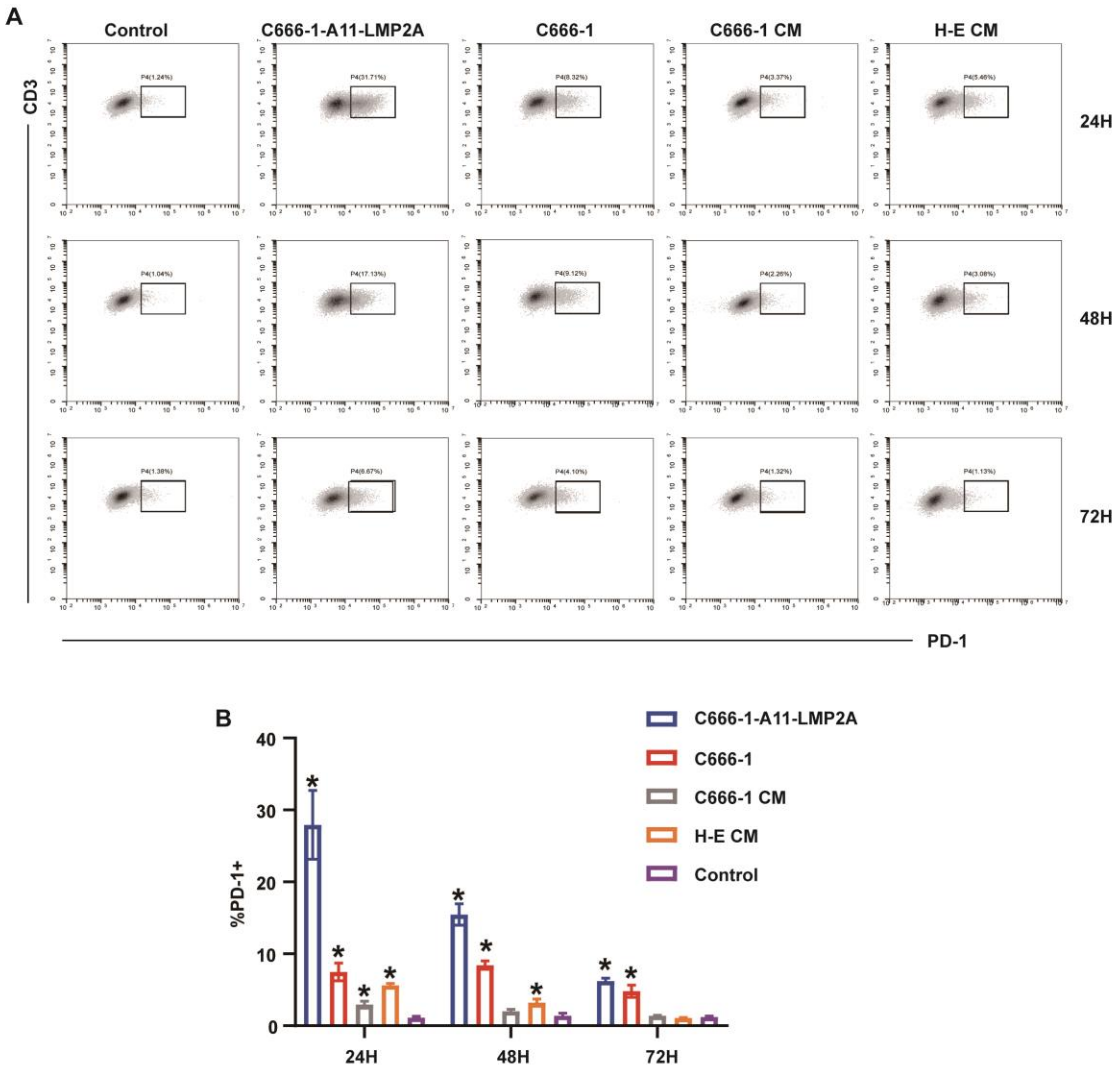

Supplementary Fig. 3. Expression of PD-1 on HUVECs after co-culture with C666-1-A11-LMP2A, C666-1, C666-1 CM, and H-E CM for 24h, 48h, and 72h, respectively. a) Representative images of Flow cytometry. **b)** Statistical plot of flow cytometry results. Data are mean ± SD, n=3, two-tailed Student's t test. *$P < 0.05$.

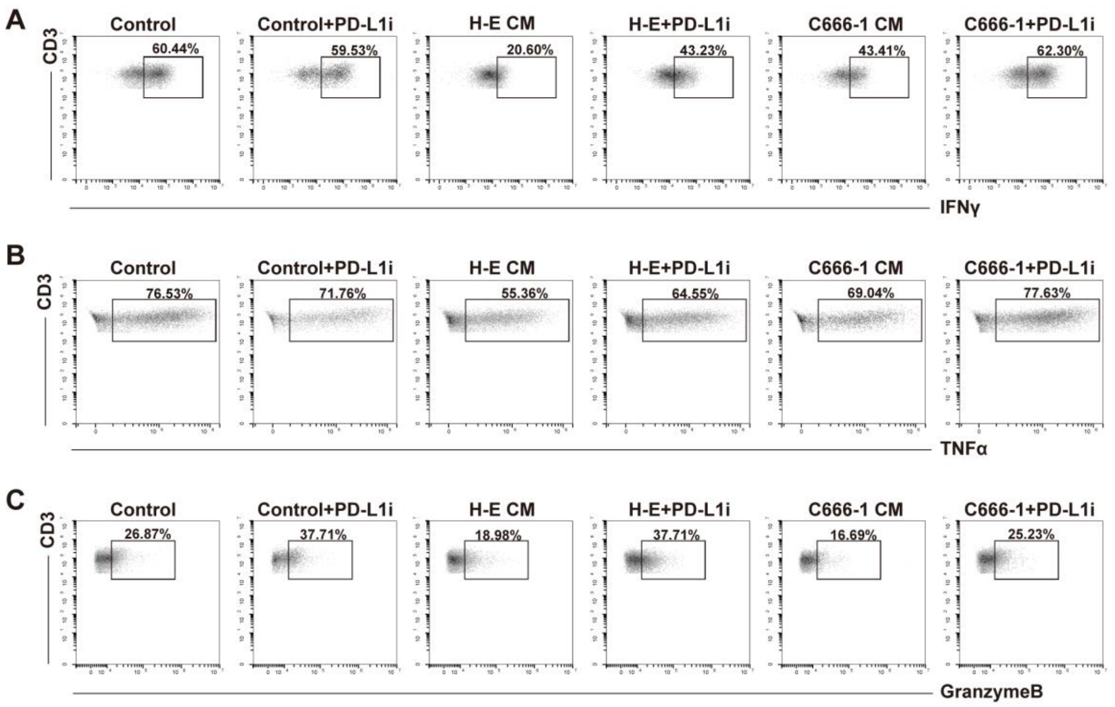

Supplementary Fig. 4. Representative images of flow cytometry of cytokines in TCR-T cells after different treatments. a) Representative images of flow cytometry of IFN-γ in TCR-T cells after different treatments. **b)** Representative images of flow cytometry of TNF-α in TCR-T cells after different treatments. **c)** Representative images of flow cytometry of GranzymeB in TCR-T cells after different treatments. *Control* indicated TCR-T co-incubated with control HUVECs, *H-E* indicated TCR-T co-incubated with HK1-EBV CM-treated HUVECs, C666-1 indicated TCR-T co-incubated with C666-1 CM-treated HUVECs, and PD-L1i indicated incorporation of PD-L1 inhibitor.

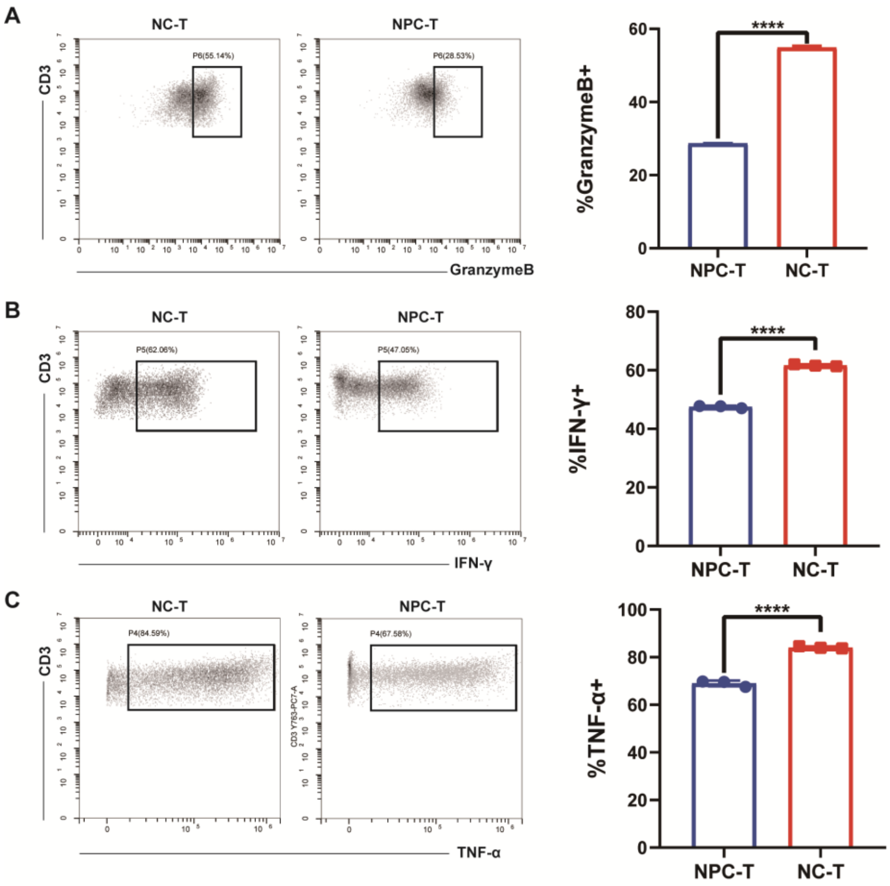

Supplementary Fig. 5. Representative images of flow cytometry and statistical plots of cytokines in NC-T cells and NPC-T cells after co-incubation with CM-HUVECs. a) Representative images of flow cytometry and statistical plot of GranzymeB in NC-T cells and NPC-T cells after co-incubation with CM-HUVECs. **b)** Representative images of flow cytometry and statistical plot of IFN-γ in NC-T cells and NPC-T cells after co-incubation with CM-HUVECs. **c)** Representative images of flow cytometry and statistical plot of TNF-α in NC-T cells and NPC-T cells after co-incubation with CM-HUVECs. Data are mean ± SD, n=3, two-tailed Student's t test, ****P < 0.0001.

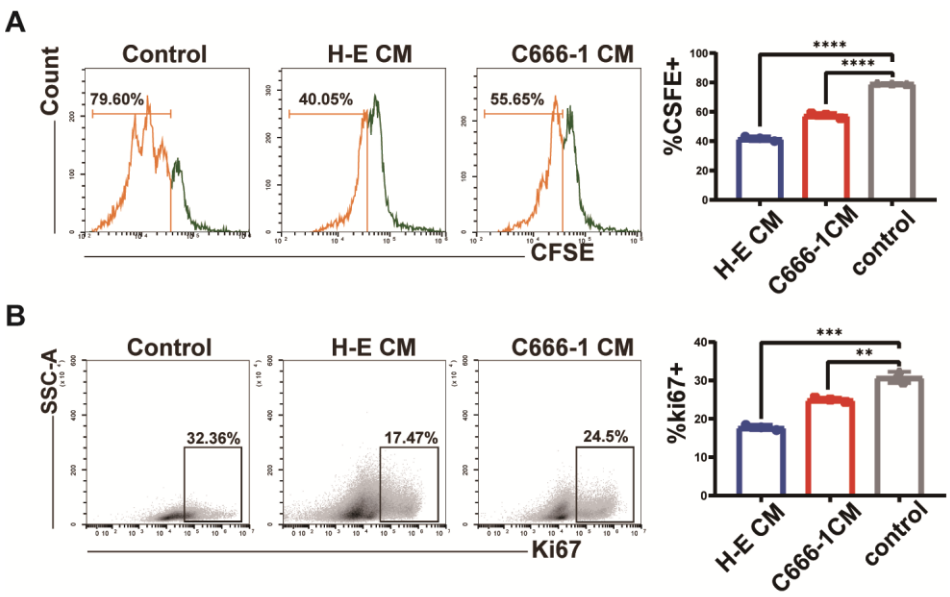

Supplementary Fig. 6. Effect of CM-treated versus untreated HUVECs on the proliferative capacity of TCR-T cells. a, b) CSFE staining **a** and Ki67 flow cytometry **b** results suggested that CM-treated HUVECs inhibited the proliferative capacity of TCR-T cells. *Control* indicated untreated HUVECs. *H-E CM* indicated H-E CM-treated HUVECs. *C666-1 CM* indicated C666-1 CM-treated HUVECs. Data are mean ± SD, n=3, two-tailed Student's t test. ***P* < 0.01, ****P* < 0.001, *****P* < 0.0001.

Supplementary Table 1. The AUCs of classification models for predicting OS and PFS of patients with NPC.

OS		PFS	
**Classification models**	**AUC**	**Classification models**	**AUC**
**TEC-PD-L1**	0.7733 (95%*CI* [0.7055-0.8412])	**TEC-PD-L1**	0.6981 (95%*CI* [0.6305-0.7657])
**TC-PD-L1**	0.5927 (95%*CI* [0.5150-0.6705])	**TC-PD-L1**	0.5695 (95%*CI* [0.4974-0.6417])
**Dual-scoring**	0.7123 (95%*CI* [0.6445-0.7800])	**Dual-scoring**	0.6448 (95%*CI* [0.5739-0.7156])
