# Supplementary material for: Tumor vascular endothelial cells promote immune escape by upregulating PD-L1 expression via crosstalk between NF-κB and STAT3 signaling pathways in nasopharyngeal carcinoma
Source: Cell Death Dis. 2025 Feb 25;16(1):129. doi: 10.1038/s41419-025-07444-z (PMC11861260; doi:10.1038/s41419-025-07444-z)
Supplement: Supplementary file 2 — Supplementary material [file 41419_2025_7444_MOESM2_ESM.pdf]

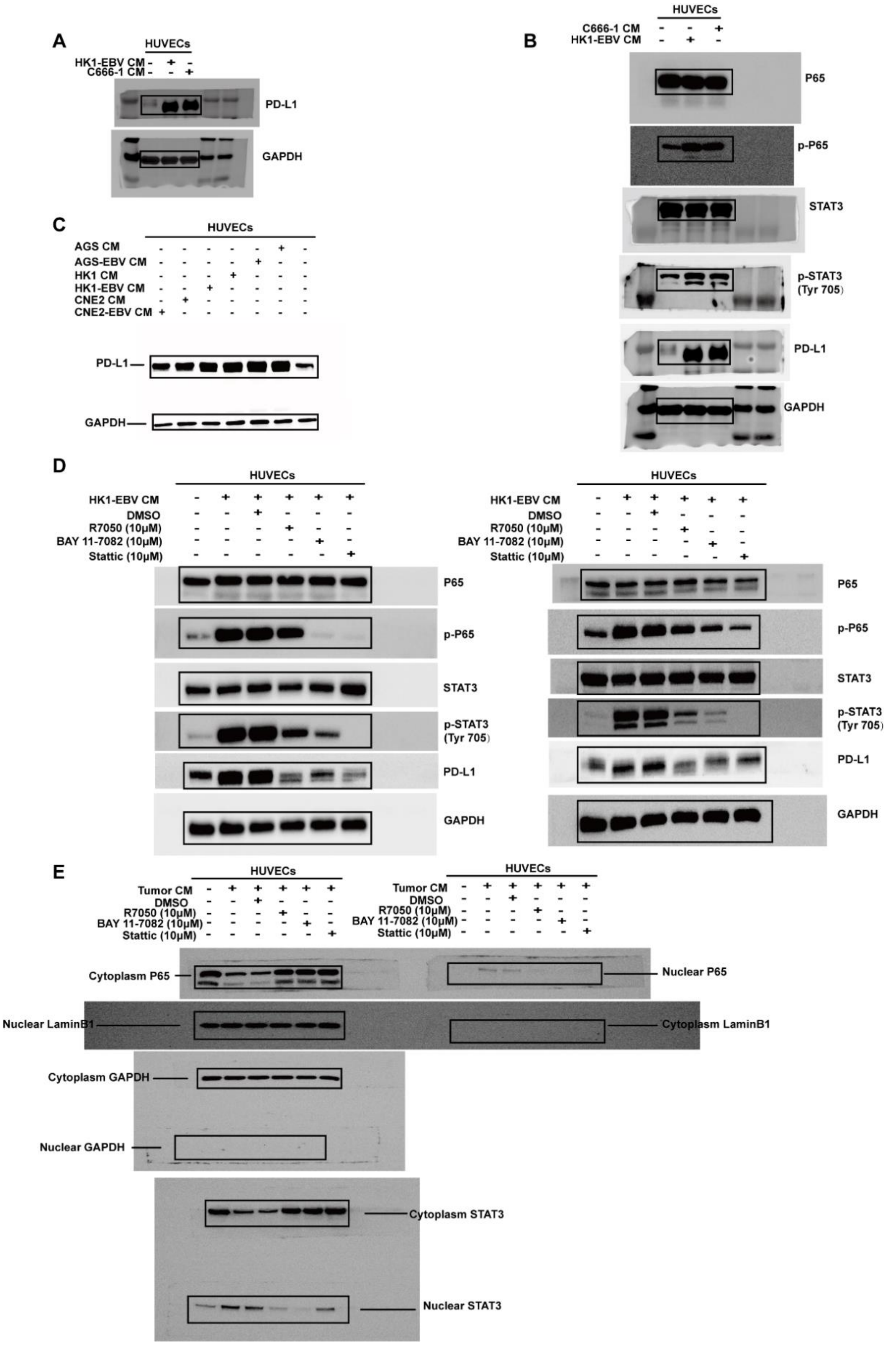

Uncropped blots presented in the figures. a) Immunoblots of cropped blots as shown in Figure 1C. **b)** Immunoblots of cropped blots as shown in Figure 5C. **c)** Immunoblots of cropped blots as shown in Figure S1. **d)** Immunoblots of cropped blots as shown in Figure 5D. **e)** Immunoblots of cropped blots as shown in Figure 5E.
